# On the brink of extinction: a new freshwater amphipod *Jesogammarusacalceolus* (Anisogammaridae) from Japan

**DOI:** 10.3897/zookeys.1065.71687

**Published:** 2021-10-26

**Authors:** Ko Tomikawa, Naoya Kimura

**Affiliations:** 1 Hiroshima University, Graduate School of Humanities and Social Sciences, 1-1-1 Kagamiyama, Higashihiroshima, Hiroshima, 739-8524, Japan Hiroshima University Hiroshima Japan; 2 Tokiwazaka 1-7-18, Hirosaki, Aomori, 036-8263, Japan unaffiliated Hirosaki Japan

**Keywords:** Ancestral state reconstruction, molecular phylogeny, systematics

## Abstract

Freshwater habitats, especially cold springs, are environments in which the risk of extinction faced by organisms remains high due to human activities. To conserve endangered species, it is important to describe and name them. Here, a new, endangered freshwater anisogammarid amphipod species, Jesogammarus (Jesogammarus) acalceolus**sp. nov.**, found in a spring in Aomori Prefecture, Japan, is described which is potentially the sole remaining habitat of this species. Both morphological and molecular phylogenetic results strongly support the nesting of the new species within *Jesogammarus*. Jesogammarus (J.) acalceolus**sp. nov.** is the first species of genus *Jesogammarus* that was found to lack a calceolus, a sensory organ located on male antenna 2. Thus, the diagnostic criteria for this genus required amendment. A reconstruction of ancestral calceoli, based on a molecular phylogenetic tree, revealed that the common ancestor of *Jesogammarus* possessed calceoli, which were secondarily lost in J. (J.) acalceolus**sp. nov.** Our results indicate that this new species, which is key to clarifying the evolution of the calceolus, is of high conservation significance.

## Introduction

Fresh water is indispensable to human life. It is also an important habitat for many aquatic organisms. Fresh water accounts for ca. 2.5% of all water on Earth (Lehner and Döll 2004). Approximately 9.5% of all known species live in fresh water (Balian et al. 2008). Deterioration of freshwater environments due to human activities remains a worldwide issue (Martínuzzí et al. 2014; Reid et al. 2019). Species inhabiting freshwater habitats are reported to be at a greater risk of extinction compared to marine and terrestrial species (Dudgeon et al. 2006; Collen et al. 2009, 2014).

Spring water is ground water that collects in soil due to rain and snow in mountainous areas. Recently, deterioration of spring water environments, leading to the depletion of spring water, caused by an inflow of domestic drainage and agricultural chemicals. Additionally, excessive pumping of groundwater for drinking and agricultural purposes has become an issue of worldwide proportions. Therefore, of the species inhabiting freshwater habitats, those that depend on spring water are considered to be at an even higher risk of extinction (Fluker et al. 2010). However, currently available taxonomic data on invertebrates inhabiting spring water appear to be insufficient, with many species remaining undescribed (Murphy et al. 2009). Although the discovery rate of species appears to be increasing, many species go unrecognized before becoming extinct (Mora et al. 2011). Thus, conducting taxonomic studies as well as naming and describing species is essential for conserving endangered species (Stork 1993; McKinney 1999; Giam et al. 2012; Coleman 2015; Costello et al. 2015).

The order Amphipoda comprises peracarid crustaceans belonging to the class Malacostraca. Of the more than 10,000 amphipod species that have been described globally, ca. 20% occur in freshwater (Väinölä et al. 2008; Horton et al. 2021). Freshwater amphipods generally prefer cool environments (Väinölä et al. 2008), and cold spring water and flowing spring water are the best habitats for them. Springs in the Japanese archipelago reportedly harbour diverse endemic amphipods (Tomikawa and Morino 2003; Tomikawa et al. 2003; Tomikawa 2017). The anisogammarid genus, *Jesogammarus* Bousfield, 1979, is the most diverse group among Japanese freshwater amphipods. *Jesogammarus* was established by Bousfield (Bousfield 1979), with *Anisogammarusjesoensis* Schellenberg, 1937 as the type species. In the same paper as that which described this type species, Bousfield established *Annanogammarus* Bousfield, 1979 and *Ramellogammarus* Bousfield, 1979 with *Gammarusannandalei* Tattersall, 1922 and *Gammarusramellus* Weckel, 1907 as type species, respectively. *Annanogammarus* was later classified as a subgenus under *Jesogammarus* (Morino 1985). At present, *Jesogammarus* is known to contain 22 species from the Japanese Archipelago, the Korean Peninsula, and the Chinese mainland (Tomikawa et al. 2017). *Jesogammarus* is morphologically similar to *Ramellogammarus*, which is endemic to North American coastal fresh waters; these genera are considered to be closely related (Bousfield 1979, 1981). The former is distinguished from the latter mainly by having an antennal sensory organ termed the calceolus (Morino 1985; Bousfield and Morino 1992). However, though molecular phylogenetic studies have been conducted previously on Anisogammaridae, the phylogenetic relationship between *Jesogammarus* and *Ramellogammarus* has not yet been fully clarified (Macdonald III 2005; Tomikawa et al. 2010; Li et al. 2020).

**Figure 1.**
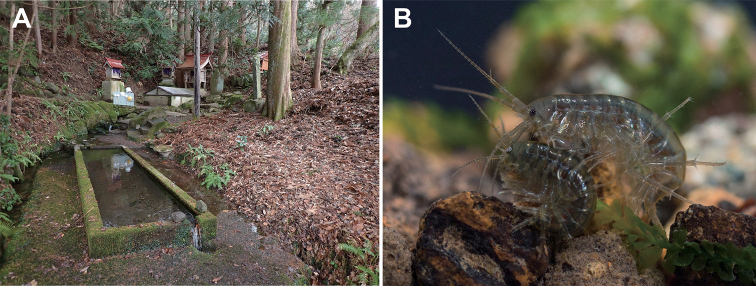
Habitat and live specimens of Jesogammarus (Jesogammarus) acalceolus sp. nov. **A** the type locality, Haguro Shrine Spring, Hirosaki, Aomori Prefecture, Japan **B** mate guarding pair, male is upper and female is lower, photographed by Ryu Uchiyama.

Recently, a population of *Jesogammarus* species, lacks a calceolus on male antenna 2, was found in a spring in the Aomori Prefecture of Japan, which is potentially the sole remaining habitat of this species (Fig. 1). We describe this species as J. (J.) acalceolus sp. nov. Describing and naming this species, as have been done here, can be considered important first steps leading to its conservation. In addition, we investigated the evolution of calceoli in *Jesogammarus* species with molecular phylogenetic analyses and ancestral state reconstruction.

## Materials and methods

### Sample collection

Specimens of J. (J.) acalceolus sp. nov. were collected from Haguro Shrine Spring, Hirosaki, Aomori Prefecture, Japan (40.6153°N, 140.3854°E). Amphipods were collected by a fine-mesh hand net from fallen leaves and mosses. Specimens were fixed in 99% ethanol on the site.

### Morphological observation

Appendages of the examined amphipods were dissected using needles under a stereomicroscope (Olympus SZX7) and mounted in gum-chloral medium on glass slides. Prepared specimens were examined by a light microscope (Nikon Eclipse Ni) and illustrated using the aid of a camera lucida attached to the light microscope. The body length was measured from the tip of the rostrum to the base of the telson along the dorsal curvature to the nearest 0.1 mm following Tomikawa et al. (2017). The specimens have been deposited in the Tsukuba Collection Center of the National Museum of Nature and Science, Tokyo (NSMT).

### PCR and DNA sequencing

Genomic DNA was extracted from the pleopod muscle of the specimens following procedures detailed by Tomikawa et al. (2014). The primer sets for PCR and cycle sequencing reactions used in this study were as follows: for 28S rRNA (28S), 28SF and 28SR (Tomikawa et al. 2012); for cytochrome c oxidase subunit I (COI), Am-COI-H and Am-COI-T (Tomikawa 2015); and for 16S rRNA (16S), 16STf (Macdonald III 2005) and 16Sbr (Palumbi 1996). PCR and DNA sequencing were performed using the method detailed by Tomikawa (2015). The newly obtained DNA sequence has been deposited in the International Nucleotide Sequence Database Collaboration (INSDC) through the DNA Data Bank of Japan (DDBJ) (Table 1).

**Table 1.** Samples used for molecular analyses with voucher/isolate number, collection locality, and NCBI GenBank accession number. Sequences marked with an asterisk (*) were obtained for the first time in this study.

Species	Voucher or isolate #	Locality	NCBI GenBank acc. nos.		
			28S rRNA	COI	16S rRNA
*Anisogammaruspugettensis*	G1500	Akkeshi Bay, Hokkaido, Japan	LC624749*	LC624757*	LC624742*
*Barrowgammarusmacginitiei*	G37	Akkeshi Bay, Hokkaido, Japan	LC624750*	LC624758*	LC624743*
*Eogammaruskygi*	G1	Naibetsu River, Hokkaido, Japan	LC214759	LC052229	LC052250
*E.possjeticus*	G3	Lake Akkeshi, Hokkaido, Japan	LC214760*	LC052230	LC052251
Jesogammarus (Annanogammarus) annandalei	G1162	Lake Biwa, Shiga, Japan	LC214786	LC052248	LC052269
J. (A.) debilis	IZCAS-I-A0325	Fangshan, Beijing, China	EF582997		EF582846
J. (A.) fluvialis	G83	Samegai, Shiga, Japan	LC214766	LC052236	LC052257
J. (A.) koreaensis	G1376	Deoksin-ri, Onsan-eup, Ulju-gun, Ulsan, Korea	LC624751*	LC624759*	
J. (A.) naritai	G1167	Lake Biwa, Shiga, Japan	LC214787	LC052249	LC052270
J. (A.) suwaensis	G88	Lake Suwa, Nagano, Japan	LC214767	LC052237	LC052258
**Jesogammarus (Jesogammarus) acalceolus sp. nov.**	NSMT-Cr 29008 (G1625)	Haguro Shrine Spring, Aomori, Japan	LC624752*	LC624760*	LC624744*
**J. (J.) acalceolus sp. nov.**	NSMT-Cr 29005 (G1845)	Haguro Shrine Spring, Aomori, Japan	LC624753*	LC624761*	LC624745*
J. (J.) bousfieldi	KUZ Z1799	Aburato, Tsuruoka, Yamagata, Japan	LC214778	LC214538	LC214795
J. (J.) fujinoi	G17	Yamagata, Japan	LC214762	LC052232	LC052253
J. (J.) hebeiensis	IZCAS-I-A0294	Yanqing, Beijing, China	EF582998		EF582847
J. (J.) hinumensis	G52	Lake Hinuma, Ibaraki, Japan	LC214765	LC052235	LC052256
J. (J.) hokurikuensis	G1838	Shimizucho, Fukui, Japan	LC624754*	LC624762*	LC624746*
J. (J.) ikiensis	G515	Iki, Nagasaki, Japan	LC214772	LC052242	LC052263
J. (J.) jesoensis	G164	Sapporo, Hokkaido, Japan	LC214769	LC052239	LC052260
J. (J.) mikadoi	G13	Rokugo, Akita, Japan	LC214761	LC052231	LC052252
J. (J.) paucisetulosus	G1037	Mito, Ibaraki, Japan	LC214780	LC052247	LC052268
J. (J.) shonaiensis	G192	Sakata, Yamagata, Japan	LC214770	LC052240	LC052261
J. (J.) spinopalpus	G32	Onjuku, Chiba Prefecture, Japan	LC214763	LC052233	LC052254
J. (J.) uchiyamaryui	KUZ Z1803	Tanie River, Iki, Nagasaki, Japan	LC214773	LC214533	LC214790
*Ramellogammarusoregonensis*	G1537	Willamette River, Corvallis, Oregon, USA	LC624755*		
*R.similimanus*	G1540	Alice Springs, Portland, Oregon, USA	LC624756*		
*Spasskogammarusspasskii*	G35	Akkeshi Bay, Hokkaido, Japan	LC214764*	LC052234	LC052255
*Gammarusmukudai*	G858	Iki, Nagasaki, Japan	AB893234	LC624763*	LC624747*
*G.nipponensis*	G797	Kiyotaki, Kyoto, Japan	AB893232	LC624764*	LC624748*

### Molecular phylogenetic analyses

The phylogenetic analyses were conducted based on sequences of nuclear 28S rRNA and mitochondrial COI and 16S rRNA genes. The alignment of COI was trivial, as no indels were observed. The sequences of 28S and 16S were aligned using the Muscle algorithm implemented in MEGA X (Kumar et al. 2018). Phylogenetic relationships were reconstructed via Maximum Likelihood (ML) and Bayesian Inference (BI). The best evolutionary models were selected based on the corrected Akaike Information Criterion (AIC) for ML and Bayesian Information Criterion (BIC) for BI using MEGA X (Kumar et al. 2018). ML phylogenies were conducted using MEGA X (Kumar et al. 2018) under the substitution model GTR+G+I, and 1,000 bootstrap replications (Felsenstein 1985) were performed to estimate statistical support for branching patterns. BI analyses were estimated using MrBayes v3.2.6 (Ronquist et al. 2012) under the substitution model GTR+G+I, with Markov chains of 10 million generations. Parameter estimates and convergence were checked using Tracer v1.7.1 (Rambaut et al. 2018), and the first 1 million trees were discarded as burn-in. Two gammarid species, *Gammarusmukudai* Tomikawa, Soh, Kobayashi & Yamaguchi, 2014 and *G.nipponensis* Uéno, 1966, were included in the analyses as outgroup taxa.

### Ancestral state reconstruction

**Figure 2.**
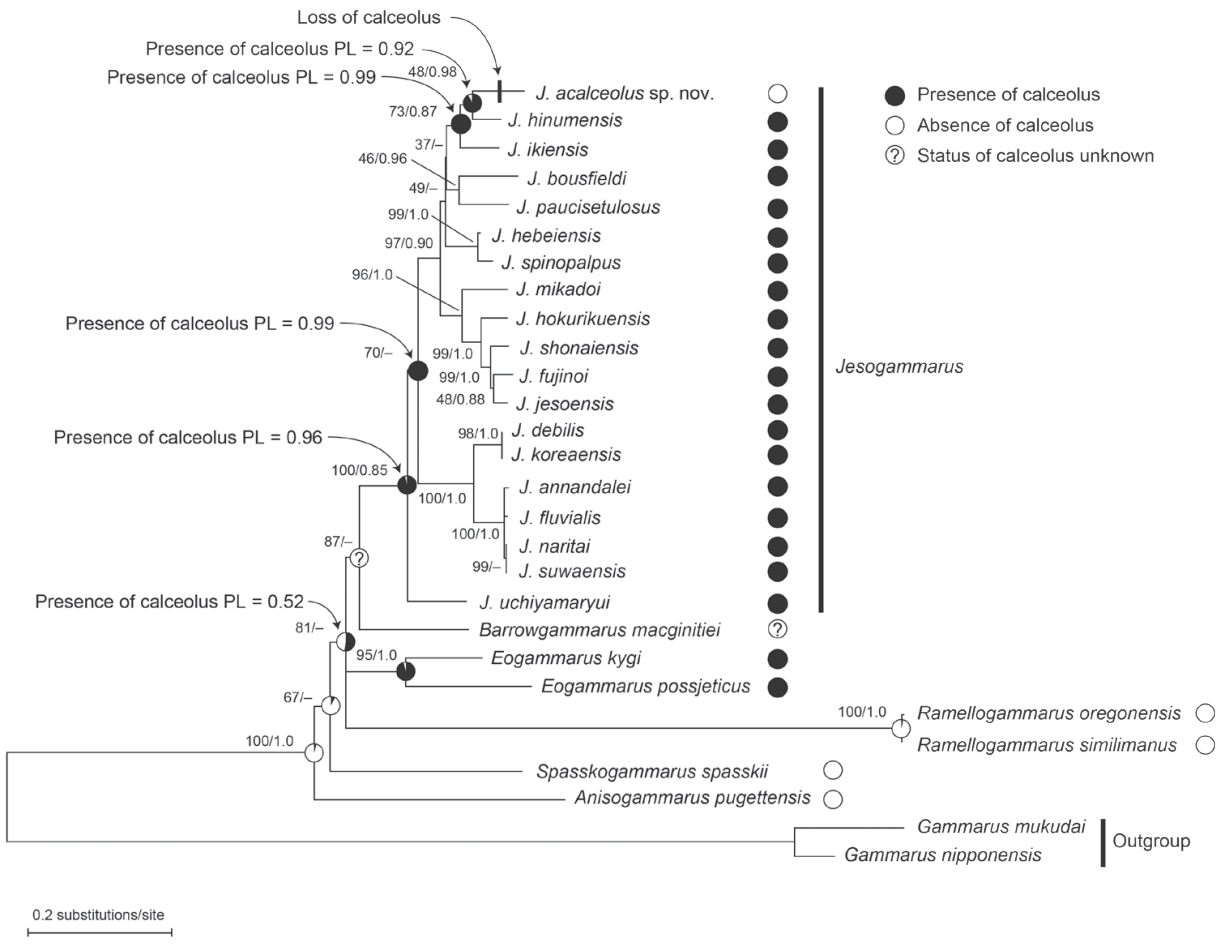
Maximum likelihood tree and ancestral state reconstructions for calceolus on male antenna 2. Filled circles at each species represent states of habitat; pie charts at internal nodes present proportional likelihoods of reconstruction. Key nodes are labelled with the proportional likelihood of the presence or absence of the calceolus on male antenna 2, which was reconstructed as most likely to be at that node.

The ancestral states of the calceolus on male antenna 2 were reconstructed on the tree (Fig. 2) via the likelihood model using Mesquite v3.61 (Maddison and Maddison 2019). The Markov K-state 1 parameter model was used for likelihood reconstruction at each ancestral node with equal probability for all particular character state changes.

## Results

### Molecular phylogenetic analyses

The monophyly of *Jesogammarus* was inferred with maximum (100% bootstrap support [BS]) and relatively low (0.85 posterior probability [PP]) support values in the maximum likelihood (ML) and Bayesian inference tree (BI) trees, respectively (Fig. 2). Although *Jesogammarus* formed a sister group with *Barrowgammarus* Bousfield, 1979 (87% BS), their relationship was not supported by BI analyses. The new species collected in this study, J. (J.) acalceolus, was nested within *Jesogammarus* and clustered with J. (J.) hinumensis Morino, 1993 and J. (J.) ikiensis Tomikawa, 2015. In this study, the phylogenetic position of J. (A.) koreaensis Lee & Seo, 1990 was also clarified for the first time: this species formed a sister group with J. (A.) debilis Hou & Li, 2005, with high support values (98% BS, 1.0 PP). Of the 22 species of *Jesogammarus*, 20, excluding J. (J.) fontanus Hou & Li, 2004 and J. (J.) ilhoii Lee & Seo, 1992, were included in the molecular phylogenetic analyses of this study.

### Ancestral state reconstruction

The likelihood reconstruction (Fig. 2) demonstrated that the calceolus on male antenna 2 was an ancestral character state of the most recent common ancestor (MRCA) of the *Jesogammarus* species, with 0.96 proportional likelihood (PL). The character state of the MRCA of J. (J.) acalceolus sp. nov. + J. (J.) hinumensis and J. (J.) acalceolus sp. nov. + J. (J.) hinumensis + J. (J.) ikiensis was the presence of calceolus, with 0.92 and 0.99 PL, respectively. The character state of the MRCA of *Barrowgammarus* + *Eogammarus* + *Jesogammarus* + *Ramellogammarus* was the presence of calceolus, with 0.52 PL.

### Taxonomic account

#### Family Anisogammaridae Bousfield, 1977

##### Genus Jesogammarus

Taxon classificationAnimaliaAmphipodaAnisogammaridae

Bousfield, 1979

FE034E58-405A-5A31-AD22-2B8613EF2E2B

###### Type species.

*Anisogammarusjesoensis* Schellenberg, 1937

###### Diagnosis.

Pleonites not carinate dorsally, with slender and robust setae (robust setae often lacking). Dorsal margins of urosomites with 4 (3), 4 (2), 2 (4) clusters of setae or single robust seta; urosomite 2 without prominent median tooth. Antenna 1 longer than antenna 2; article 1 of peduncle subequal to or slightly longer than article 2. Male antenna 2, flagellum with or without calceoli. Maxilla 1, palp article 1 without setae. Female gnathopods 1 and 2 strongly dissimilar. Coxal gills on gnathopod 2 and pereopods 3–7, gills 2–5 each with 2 accessory lobes, gills 6 and 7 each with 1 accessory lobe. Uropods 1 and 2, rami extending beyond peduncle of uropod 3. Uropod 3, inner ramus not longer than 0.4 times of that of outer ramus; terminal article distinct. Brood plate 2 of female broadly expanded anteroproximally.

###### Remarks.

The presence of a calceolus on the flagellum of male antenna 2 is a major diagnostic feature of *Jesogammarus*, which distinguishes it from *Ramellogammarus* (Bousfield 1979; Morino 1985). However, the discovery of the new species, *J.acalceolus*, which lacks a calceolus, indicated that the calceolus was not critical for diagnosis. The genus *Jesogammarus* is distinguishable from *Ramellogammarus* by the dissimilar female gnathopods 1 and 2 and the expanded brood plates of the female. The genus *Jesogammarus* shares a similar coxal gill type with marine *Locustogammarus* Bousfield, 1979 and *Spasskogammarus* Bousfield, 1979 but differs from these two genera in terms of the following features (features of *Locustogammarus* and *Spasskogammarus* in parentheses): from *Locustogammarus*, in terms of longer antenna 1 than antenna 2 (subequal in *Locustogammarus*), dissimilar female gnathopods 1 and 2 (similar in *Locustogammarus*), uropods 1 and 2 with rami extending beyond the peduncle of uropod 3 (not extending in *Locustogammarus*), and a distinct terminal article of uropod 3 (very small in *Locustogammarus*); from *Spasskogammarus*, in terms of dorsal margins of pleonites with slender setae (lacking in *Spasskogammarus*), longer antenna 1 than antenna 2 (subequal in *Spasskogammarus*), and slender pereopods 5–7 (short in *Spasskogammarus*).

##### Jesogammarus (J.) acalceolus sp. nov.

Taxon classificationAnimaliaAmphipodaAnisogammaridae

1DB82211-EAD7-5024-97B6-95672F0BEFCF

http://zoobank.org/43EABC71-3F5A-48ED-9982-6320B94C6CAC

**Figure 3.**
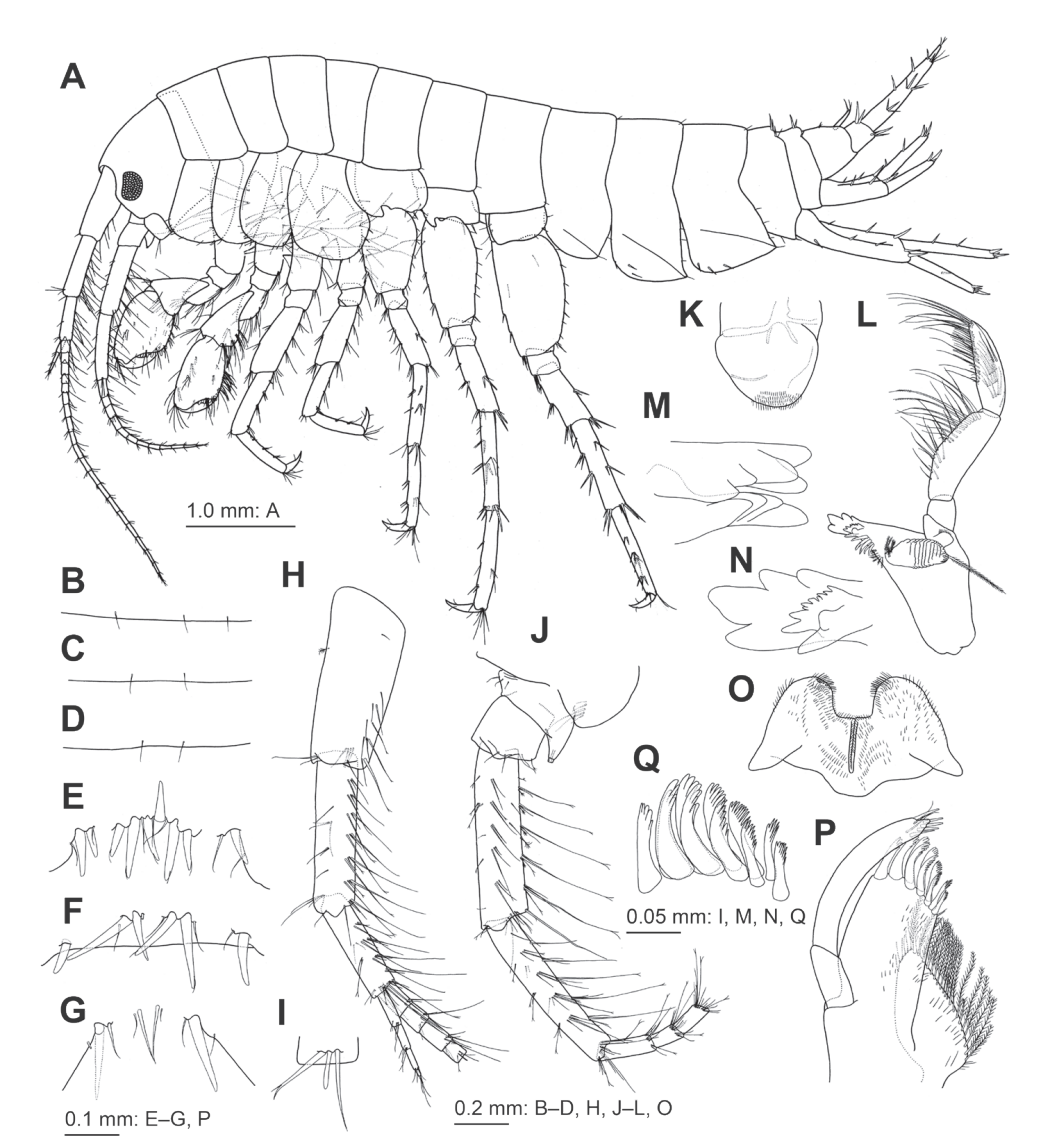
Jesogammarus (Jesogammarus) acalceolus sp. nov., male (7.4 mm), NSMT-Cr 29003 **A** habitus, lateral view **B–D** dorsal margins of pleonites 1–3, respectively, dorsal views **E–G** dorsal margins of urosomites 1–3, respectively, dorsal views **H** peduncular articles 1–3, accessory flagellum, and flagellar articles 1–4 of antenna 1, medial view **I** aesthetasc and associate setae on the flagellum of antenna 1, medial view **J** peduncular articles 1–5 and flagellar articles 1–3 of antenna 2, medial view **K** upper lip, posterior view **L** right mandible, medial view **M–N** incisor and lacinia mobilis of left and right mandibles, medial views **O** lower lip, ventral view **P** maxilla 1, medial view **Q** serrate robust setae on outer plate of maxilla 1, medial view.

**Figure 4.**
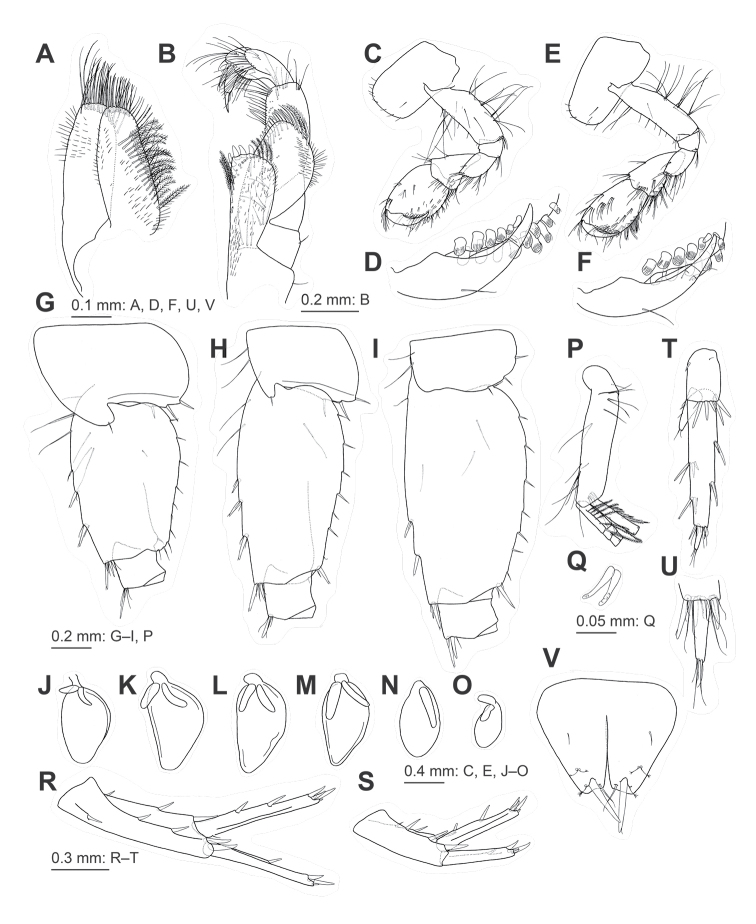
Jesogammarus (Jesogammarus) acalceolus sp. nov., male (7.4 mm), NSMT-Cr 29003 **A** maxilla 2, medial view **B** maxilliped, dorsal view **C** gnathopod 1, medial view **D** palmar margin of propodus and dactylus of gnathopod 1, medial view, some setae omitted **E** gnathopod 2, medial view **F** palmar margin of propodus and dactylus of gnathopod 2, medial view, some setae omitted **G–I** coxa-ischium of pereopods 5–7, respectively, lateral views **J–O** coxal gills on gnathopod 2–pereopod 7, respectively, lateral views **P** pleopod 1, lateral view, distal parts of rami omitted **Q** retinacula on peduncle of pleopod 1, lateral view **R–S** uropods 1–2, respectively, dorsal views **T** uropod 3, ventral view **U** distal part of proximal article and terminal article of outer ramus of uropod 3, ventral view **V** telson, dorsal view.

**Figure 5.**
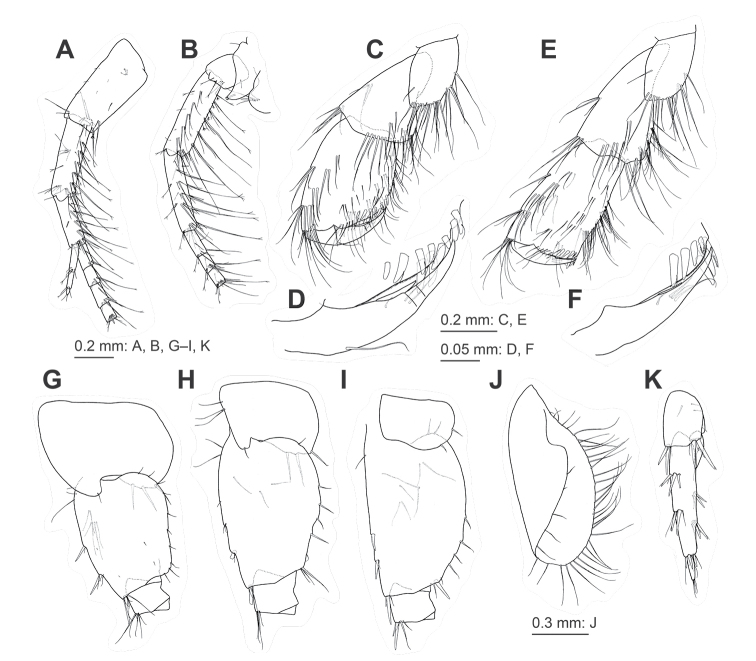
Jesogammarus (Jesogammarus) acalceolus sp. nov., female (7.3 mm), NSMT-Cr 29004 **A** peduncular articles 1–3, accessory flagellum, and flagellar articles 1–4 of antenna 1, medial view **B** peduncular articles 1–5 and flagellar articles 1–3 of antenna 2, medial view **C** ischium-dactylus of gnathopod 1, medial view **D** palmar margin of propodus and dactylus of gnathopod 1, medial view, some setae omitted **E** ischium-dactylus of gnathopod 2, medial view **F** palmar margin of propodus and dactylus of gnathopod 2, medial view, some setae omitted **G–I** coxa-ischium of pereopods 5–7, respectively, lateral views **J** brood plate on gnathopod 2, lateral view **K** uropod 3, ventral view.

[New Japanese name: Shitsuko-yokoebi] Figures 1B , 3 , 4 , 5

###### Material examined.

***Holotype***: male (7.4 mm, NSMT-Cr 29003), Haguro Shrine Spring, Hirosaki, Aomori Prefecture, Japan (40.6153°N, 140.3854°E), collected by A. Ohtaka, N. Kimura, and K. Tomikawa on 10 December 2020. ***Paratypes***: two females (7.3 mm, NSMT-Cr 29004; 6.7 mm, NSMT-Cr 29005 [G1845]), two male (7.7 mm, NSMT-Cr 29006; 7.5 mm, NSMT-Cr 29007 [G1844]), data same as for the holotype; male (6.8 mm, NSMT-Cr 29008 [G1625]), same locality of the holotype, collected by A. Ohtaka on 23 December 2018; 3 males (7.3–7.6 mm, NSMT-Cr 29009) and three females (6.4–7.3 mm, NSMT-Cr 29009), same locality of the holotype, collected by A. Ohtaka on 17 June 2018; 3 males (5.8–8.0 mm, NSMT-Cr 29009) and three females (5.3–6.4 mm, NSMT-Cr 29009), same locality of the holotype, collected by N. Kimura on 23 December 2018; seven males (7.6–8.8 mm, NSMT-Cr 29009) and three females (5.6–6.6 mm, NSMT-Cr 29009), same locality of the holotype, collected by N. Kimura on 10 December 2020; 10 males (6.9–9.9 mm, NSMT-Cr 29009) and 11 females (5.9–8.3 mm, NSMT-Cr 29009), same locality of the holotype, collected by N. Kimura on 12 December 2020.

###### Diagnosis.

Dorsal surface of pereonites smooth. Pleonites 1–3 each with fewer than three dorsal setae. Antenna 1 without robust seta on posterodistal corner of peduncular article 1. Male antenna 2 without calceoli. Mandible with palp article 1 lacking setae. Uropod 3 without plumose setae on outer ramus.

###### Description.

**Male** [7.4 mm, NSMT-Cr 29003].

***Body*.** Head (Fig. 3), rostrum short; lateral cephalic lobe with ventral margin weakly concave; antennal sinus rounded; eyes small, subreniform, major axis 0.3 × head height. Pereonites, dorsal surfaces smooth (Fig. 3). Pleonites 1–3 (Fig. 3B–D), dorsal margins each with three, two, and two setae. Epimeral plate 1 with rounded posterior margin bearing seta, seta on posteroventral corner (Fig. 3A); epimeral plate 2 with posterior margin almost straight bearing three setae, seta on weakly produced posteroventral corner, two and one robust setae on ventral margin and submargin, respectively (Fig. 3A); epimeral plate 3 with posterior margin almost straight bearing three setae, seta on quadrate posteroventral corner, three robust setae on ventral margin (Fig. 3A). Urosomite 1 (Fig. 3E) with dorsal margin bearing a pair of lateral robust setae and a middle cluster of robust setae; urosomite 2 (Fig. 3F) with dorsal margin bearing a pair of lateral robust setae and clusters of robust setae; urosomite 3 (Fig. 3G) with dorsal margin bearing a pair of robust setae.

***Antennae*.** Antenna 1 (Fig. 3H) 0.6 ×length of body; length ratio of peduncular articles 1–3 in 1.0 : 0.9 : 0.6; peduncular article 1 with posterodistal corner lacking robust seta, posterior margin with three pairs of setae and single seta; peduncular article 2 with posterior margin bearing six clusters of setae; peduncular article 3 with posterior margin bearing four clusters of setae; accessory flagellum comprising four articles; primary flagellum comprising 20 articulate, aesthetasc on each article. Antenna 2 (Fig. 3J) 0.7 × length of antenna 1; article 4 of peduncular 1.1 × article 5; peduncular articles 4 and 5 with posterior margins each bearing five setal clusters; flagellum comprising 12 articles, calceoli absent.

***Mouth parts*.** Upper lip (Fig. 3K) with fine seta on rounded distal margin. Mandibles (Fig. 3L–N), left and right incisors comprising five and four teeth, respectively, left lacinia mobilis comprising four teeth, right lacinia mobilis bifid with many denticles; molar process triturative with plumose seta; left and right mandibles with seven and five blade-like setae on accessory setal rows, respectively; palp comprising 3 articles with length ratio of 1.0 : 3.3 : 2.8; article 1 of palp without setae; article 2 with 25 setae; article 3 bearing pair of setae on inner surface, three clusters of setae and single seta on outer surface. Lower lip (Fig. 3O), outer lobes broad, inner lobes indistinct. Maxilla 1 (Fig. 3P) with medial margin of inner plate bearing 20 plumose setae; eleven serrate robust setae on outer plate apically (Fig. 3Q); palp comprising 2 articles, article 1 marginally bare, apical margin of article 2 with five robust setae and two slender seta. Maxilla 2 (Fig. 4A) with inner plate bearing oblique inner row of 17 plumose setae. Maxilliped (Fig. 4B) with inner plate bearing three apical and two inner marginal robust setae; outer plate, apical margin with plumose setae and inner margin with robust setae; palp comprising four articles, inner margin and submargin of article 2 with rows of setae, article 3 bearing facial setae, slightly curved article 4 with slender nail.

***Gnathopods*.** Gnathopod 1 (Fig. 4C, D) with coxa bearing marginal setae ventrally; basis with long setae on anterior and posterior margins; length of carpus 1.4 × width, with seta on anterior margin; length of propodus 1.3 × carpus and 1.4 × width, bearing two clusters of setae on anterior margin, propodus with oblique and weakly convex palmar margin bearing six medial and ten lateral peg-like robust setae; dactylus weakly curved, as long as palmar margin. Gnathopod 2 (Fig. 4E, F) with coxa bearing marginal setae ventrally; basis with anterior and posterior margins bearing long setae; length of carpus 1.8 × width, bearing setae on anterior margin; length of propodus 1.1 × carpus and 1.6 × width, respectively, with two clusters of setae on anterior margin, propodus with oblique and weakly convex anterior margin bearing eight medial and five lateral peg-like robust setae; dactylus weakly curved, as long as palmar margin.

***Pereopods*.** Pereopods 3 and 4 (Fig. 3A) similar, coxa of pereopod 3 subrectangular with ventral setae; coxa of pereopod 4 expanded with posterior concavity, anterodistal corner and ventral margin with setae. Pereopod 5 (Figs 3A, 4G) with bilobed coxa bearing apical seta on anterior lobe, two robust setae on ventral margin of posterior lobe, posterodistal corner of posterior lobe rounded with robust seta; basis with weakly expanded posterior margin bearing setae, posterodistal corner not lobate. Pereopod 6 (Figs 3A, 4H) with bilobed coxa bearing anteroproximal setae and apical seta on anterior lobe, two robust setae on ventral margin of posterior lobe, posterodistal corner of posterior lobe quadrate with robust seta; basis with weakly expanded posterior margin bearing setae, posterodistal corner not lobate. Pereopod 7 (Figs 3A, 4I) with weakly concave coxa in ventral margin bearing setae; basis with weakly expanded posterior margin bearing setae, posterodistal corner not lobate with robust and slender setae.

***Coxal gills*** (Fig. 4J–O) with two accessory lobes on gills 2–5, posterior lobes longer than or equal to anterior ones, one accessory lobe on gills 6 and 7.

***Pleopods 1–3*** (Fig. 4P) with peduncle bearing paired retinacula (Fig. 4Q) on inner margin; inner ramus with inner basal margin bearing bifid plumose setae.

***Uropods*.** Uropod 1 (Fig. 4R) with peduncle bearing basofacial robust seta, two robust setae on inner and outer margins, one and two robust setae on inner and outer distal corners, respectively; length of inner ramus 0.8 × that of peduncle, inner margin of inner ramus with two robust setae; length of outer ramus 0.9 × that of inner ramus, inner margin of outer ramus with robust seta. Uropod 2 (Fig. 4S) with peduncle bearing two robust setae on inner and outer margins, respectively, and robust seta on inner and outer distal corners; length of inner ramus 0.9 × that of peduncle, inner margin of inner ramus with two robust seta; length of outer ramus 0.8 × that of inner ramus, inner margin of outer ramus with robust seta. Uropod 3 (Fig. 4T, U) with peduncle length 0.3 × that of outer ramus; length of inner ramus 0.3 × that of outer ramus, inner ramus with slender setae on inner margin and setae apically; outer ramus comprising two articles, proximal article with two clusters of setae on inner and outer margins, some of which robust, lacking plumose setae, length of terminal article 0.2 × that of proximal article, apical part of terminal article with simple setae.

***Telson*** (Fig. 4V) 0.8 times as long as wide, cleft for 67% of length, with robust seta and slender setae on each lobe.

###### Female

[7.3 mm, NSMT-Cr 29004].

***Antennae*.** Antenna 1 (Fig. 5A), length ratio of peduncular articles 1–3 in 1.0 : 0.8 : 0.6; peduncular article 1 with pair of setae and single seta on posterior margin; peduncular article 2 with five clusters of setae on posterior margin; accessory flagellum comprising three articles; primary flagellum comprising 17 articles. Antenna 2 (Fig. 5B) with peduncular article 4 bearing six clusters or single setae on posterior margin; peduncular article 5 with five clusters or single setae on posterior margin; flagellum comprising eleven articles, lacking calceoli.

***Gnathopods*.** Gnathopod 1 (Fig. 5C, D) with carpus bearing cluster of setae on anterior margin; length of propodus 1.2 × that of carpus and 1.5 × width; propodus with eight medial and two lateral robust setae on palmar margin. Gnathopod 2 (Fig. 5E, F) with carpus bearing cluster of setae on anterior margin; propodus and carpus approximately the same length, propodus with three medial and two lateral robust setae and one medial and one lateral pectinate robust setae on palmar margin.

***Pereopods 5–7*** with more expanded posterior margin of bases than those of male (Fig. 5G–I).

***Brood plates*** (= oostegites) (Fig. 5J) wide, with numerous setae on its margins.

***Uropod 3*** (Fig. 5K), length of peduncle 0.4 × that of outer ramus; length of inner ramus 0.2 × that of outer ramus.

###### Variations.

Although almost all specimens have a pleonite 1 with a pair of setae on the dorsal margin, a few specimens have three setae. Some specimens have a urosomite 1 with a pair of lateral robust setae and a pair of clusters of robust setae on its dorsal margin. The numbers of setal clusters on the posterior margins of the peduncular articles 1–3 of antenna 1 ranged from two to four, six or seven, and two to four, respectively. The number of setal clusters on the posterior margins of the peduncular articles 4 and 5 ranged from five or six and four or five, respectively. Some specimens have robust setae on the outer margin of the outer ramus of uropod 1 and lack robust setae on the inner margin of the outer ramus of uropod 2. Some specimens have a telson with 2 robust setae on each lobe. The number of eggs is up to 9.

###### Etymology.

The new specific name derived from the absence of calceolus.

###### Remarks.

Jesogammarus (J.) acalceolus sp. nov. differs from its congeners by lacking a calceolus on the flagellum of antenna 2 in male. This new species is similar to J. (J.) bousfieldi Tomikawa, Hanzawa & Nakano, 2017 and J. (J.) paucisetulosus Morino, 1984 in having the following features: eyes are small; antenna 1 lacks robust setae on the posterodistal corner of the peduncular article 1; antennae 1 and 2 have many long setae on the posterior margins of the peduncular articles; maxilla 1 lacks setae on the outer margin of the palp article 2; and gnathopods 1 and 2 have few setae on the ventral margins of the coxae in female. In addition to the absence of a calceolus, J. (J.) acalceolus sp. nov. is distinguished from J. (J.) bousfieldi by the pleonites 1–3 each with less than three setae on the dorsal margins (vs. more than four setae in J. (J.) bousfieldi).

###### Assessment of conservation status.

Jesogammarus (J.) acalceolus sp. nov. was found in a spring located 120 m above sea level, on the slope of the volcanic Mt. Iwaki, Aomori Prefecture, Japan. Although we conducted an intensive survey of inland waters at more than 400 sites in the Aomori Prefecture, this new species was present only in this one spring described above and not found in any others (unpublished data). In most of the freshwater habitats that were investigated, J. (J.) jesoensis Schellenberg, 1937, which is distributed in Hokkaido and northern Honshu, was present. Because J. (J.) acalceolus sp. nov. and J. (J.) jesoensis are not closely related (Fig. 2), it is expected that the current distributions of both species are a result of different evolutionary processes. As a positive aspect, the type locality of J. (J.) acalceolus sp. nov. is in the precincts of the Iwaki Haguro Shrine, built in AD 807, as a result of which this type locality has been treated with care by locals for more than 1,000 years (Sasaki 1995). Therefore, the environment of this spring has been preserved in good condition, allowing the present J. (J.) acalceolus sp. nov. population to survive. At present, this spring has an abundance of water (60 m^3^/day) (Yamamoto 1994), and its environment is stable. However, amphipods are known to be highly sensitive to chemicals, such as pesticides (Schulz 2003; Nyman et al 2013). This species inhabits only a few meters of a spring brooklet surrounded by apple plantations. Therefore, the deterioration of its habitat due to an inflow of agricultural chemicals into spring water may lead to its extinction.

### Key to species of *Jesogammarus* based on Tomikawa et al. (2017)

**Table:** 

1	Accessory lobes of coxal gills on gnathopod 2 and pereopods 3–5 well developed, both anterior and posterior lobes subequal in length or posterior lobe longer than anterior one; palmar margin of propodus of female gnathopod 2 with pectinate setae	**2** (**subgenus Jesogammarus**)
–	Accessory lobes of coxal gills on gnathopod 2 and pereopods 3–5 weakly developed, anterior and posterior lobes unequal in length, often posterior lobe rudimentary; palmar margin of propodus of female gnathopod 2 without pectinate setae	**13** (**subgenus Annanogammarus**)
2	Article 1 of mandibular palp with setae	**3**
–	Article 1 of mandibular palp without setae	**6**
3	Dorsal margin of pleonites 1–3 each with 1–2 setae; eye large; article 1 of mandibular palp with 1 robust seta; female pereopods densely setose	***J.hinumensis* Morino, 1993**
–	Dorsal margin of pleonites 1–3 each with more than 4 setae; eye small to medium; article 1 of mandibular palp with 2 or 3 robust setae; female pereopods not densely setose	**4**
4	Peduncular article 1 of antenna 1 with robust seta on posterodistal corner	***J.spinopalpus* Morino, 1985**
–	Peduncular article 1 of antenna 1 with slender seta on posterodistal corner	**5**
5	Inner ramus of uropod 3 length 1/4 × outer ramus; inner margin of outer ramus of uropod 3 with 4–6 plumose setae	***J.fontanus* Hou & Li, 2004**
–	Inner ramus of uropod 3 length 1/3 × outer ramus; inner margin of outer ramus of uropod 3 with ca. 10 plumose setae	***J.hebeiensis* Hou & Li, 2004**
6	Male antenna 2 without calceoli	***J.acalceolus* sp. nov.**
–	Male antenna 2 with calceoli	**7**
7	Dorsal margin of pereonites 1–3 each with 2 long setae	***J.mikadoi* Tomikawa, Morino & Mawatari, 2003**
–	Dorsal margin of pereonites 1–3 without setae	**8**
8	Posterodistal corner of peduncular article 1 of antenna 1 without robust seta; posterior margin of peduncular article 2 of antenna 1 with more than 5 setae and/or setal bundles; outer margin of palp article 2 of maxilla 1 without setae	**9**
–	Posterodistal corner of peduncular article 1 of antenna 1 with robust seta (occasionally lacking); posterior margin of peduncular article 2 of antenna 1 with less than 4 setae and/or setal bundles; outer margin of palp article 2 of maxilla 1 with setae	**10**
9	Dorsal margins of pleonites 1–3 each with more than 4 setae	***J.bousfieldi* Tomikawa, Nakano & Hanzawa, 2017**
–	Dorsal margins of pleonites 1–3 each with 0–3 setae	***J.paucisetulosus* Morino, 1984**
10	Accessory lobes of coxal gills on gnathopod 2 and pereopods 3–5 short and straight	***J.uchiyamaryui* Tomikawa, Nakano & Hanzawa, 2017**
–	Accessory lobes of coxal gills on gnathopod 2 and pereopods 3–5 long and curved	**11**
11	Dorsal margins of pleonites 1–3 each with 2 or 3 setae; posterior margin of peduncular article 2 of antenna 1 with 3 or 4 setae and/or setal bundls	***J.ikiensis* Tomikawa, 2015**
–	Dorsal margins of pleonites 1–3 each with more than 7 setae; posterior margin of peduncular article 2 of antenna 1 with 2 setae and/or setal bundls	**12**
12	Palmar margin of propodus of male gnathopod 2 without pectinate setae	***J.jesoensis* complex** [see Tomikawa et al. (2016)]
–	Palmar margin of propodus of male gnathopod 2 with pectinate setae	***J.ilhoii* Lee & Seo, 1992**
13	Dorsal margin of pleonite 3 with robust setae; posterior margin of peduncular articles 4 and 5 each with more than 5 long-setal bundles	***J.naritai* Morino, 1985**
–	Dorsal margin of pleonite 3 without robust setae; posterior margin of peduncular articles 4 and 5 each with less than 3 short-setal bundles	**14**
14	Posterodistal corner of bases of pereopods 5–7 with long setae	***J.annandalei* (Tattersal, 1922)**
–	Posterodistal corner of bases of pereopods 5–7 without short setae	**15**
15	Dorsal margins of pleonites 1–3 each with 2–4 setae	***J.fluvialis* Morino, 1985**
–	Dorsal margins of pleonites 1–3 each with more than 10 setae	**16**
16	Posterodistal corner of peduncular article 1 of antenna 1 with robust seta; palmar margin of propodus of female gnathopod 2 with simple setae only	***J.koreaensis* Lee & Seo, 1990**
–	Posterodistal corner of peduncular article 1 of antenna 1 without robust seta; palmar margin of propodus of female gnathopod 2 with weakly pectinate setae	***J.debilis* Hou & Li, 2005**

## Discussion

Among freshwater habitats, springs have an especially high risk of extinction of species (Fluker et al. 2010). The highly diverse genus *Jesogammarus*, which is found in spring water habitats of the Japanese Archipelago, has a sensory organ termed the calceolus on male antenna 2. We described a new endangered freshwater amphipod species, Jesogammarus (Jesogammarus) acalceolus sp. nov., found in a spring in Aomori Prefecture, Japan, which is potentially the sole remaining habitat of this species.

Although the calceolus is thought to be a sensory organ, its function and evolution are not well understood (Lincoln and Hurley 1981; Godfrey et al. 1988; Read and Williams 1990; Dunn 1998). Therefore, the discovery of J. (J.) acalceolus sp. nov., which lacks calceoli, provides important clues regarding the function and evolution of calceoli. An ancestral reconstruction of calceoli via the molecular phylogenetic tree generated during this study revealed that the common ancestor of *Jesogammarus* possessed calceoli, which were secondarily lost in J. (J.) acalceolus sp. nov. (Fig. 2). Since *Jesogammarus* carried calceoli only on the flagellum of antenna 2 of males, it is considered that calceoli have a reproductive function (Bousfield and Shih 1994). Females of amphipods lay eggs just after moulting when the exoskeleton is soft. Therefore, some species display a reproductive behaviour termed “precopula”, in which a male holds and guards a female for a couple of days till the female’s moulting and subsequent laying eggs. Dunn (1998) reported that calceoli can be used to evaluate the moulting interval of females to find suitable females for mate guarding. In this study, J. (J.) acalceolus sp. nov., the males of which lack calceoli, was also found to practice precopulatory guarding, which suggested that calceoli are not always necessary for precopulatory guarding in J. (J.) acalceolus sp. nov.

The calceolus is a typically club- or paddle-shaped structure found on the antennae of amphipods (Schmitz 1992). Although structures similar to the calceolus are also found in the antennal articles of Anaspidacea and Mysida, these are not considered to be homologous to amphipod calceoli (Bousfield and Shih 1994). Calceoli are used mainly as a taxonomic character in the higher taxa of amphipods (Lincoln and Hurley 1981; Holsinger 1992; Bousfield and Shih 1994). In Anisogammaridae, the presence or absence of calceoli is used as a genus-level taxonomic feature (Bousfield 1979). However, the molecular phylogenetic tree generated in this study confirmed that the non-calceolate species, J. (J.) acalceolus sp. nov., is nested in *Jesogammarus*, and not in *Anisogammarus*, *Ramellogammarus* or *Spasskogammarus*, the other Anisogammaridae with non-calceolate species (Fig. 2). These results indicated that the calceolus should no longer be used as a diagnostic feature of *Jesogammarus* and the genus needs to be redefined. Therefore, in this study, we have amended the diagnosis of *Jesogammarus*. In *Gammarus*, the seasonal variation of the presence or absence of calceoli was known (Karaman and Pinkster 1977), but J. (J.) acalceolus sp. nov., lacks calceoli year-round, suggesting that male antenna 2 lacking calceoli is a stable taxonomic feature.

Freshwater amphipods have low dispersal ability, and there thus exists a high tendency for endemic species to be distributed throughout each region (Tomikawa 2017). In addition, our taxonomic studies revealed a considerable presence of *Jesogammarus* fauna in the Japanese Archipelago (Tomikawa and Morino 2003; Tomikawa et al. 2003, 2017; Tomikawa 2015). For these reasons, it is unlikely that J. (J.) acalceolus sp. nov. will be found outside type localities, thereby limiting the current habitat of this species to a great extent. In the past, there have been many cold springs in Hirosaki with the type locality of this new species. However, recent, rapid urbanization has led to a depletion of such springs (Sasaki 1995). Besides, the habitat of this species may have been lost due to the disappearance of springs and/or environmental pollution caused by the use of agrochemicals, both of which were associated with apple plantations that flourished in this region. Thus, to conserve what is possibly the only remaining population of J. (J.) acalceolus sp. nov., it will be necessary to conduct further investigations into risk factors and develop a conservation plan with the cooperation of local communities and policymakers. In conclusion, our results indicate that this new species, which is key to clarifying the evolution of the calceolus, is of high conservation significance.

## Supplementary Material

XML Treatment for Jesogammarus

XML Treatment for Jesogammarus (J.) acalceolus
